# OATP1B3 (699G>A) and CYP2C9*2, *3 significantly influenced the transport and metabolism of glibenclamide and glipizide

**DOI:** 10.1038/s41598-018-36212-7

**Published:** 2018-12-24

**Authors:** Fayou Yang, Linlin Liu, Lin Chen, Mingyi Liu, Fanglan Liu, Yuqing Xiong, Xiao Hu, Chunhua Xia

**Affiliations:** 1 Clinical Pharmacology Institute, Nanchang University, Nanchang, 330006 P.R. China; 2 Nanchang Hongdu Hospital of TCM, Nanchang, 330038 P.R. China

## Abstract

Glibenclamide and glipizide show large substantial inter-individual variation in clinical efficacy, which may be resulted from the genetic differences of metabolic enzymes and transporters in individuals. This study purposed to investigate the effect of OATP1B3 and CYP2C9 genetic polymorphisms on the transport and metabolism of glibenclamide and glipizide in human. An LC-MS method was used to determine the uptake of glibenclamide and glipizide in OATP1B3, OATP1B3 (344T > G) and OATP1B3 (699G > A)-HEK293T cells and their metabolism in CYP2C9*1, *2 and *3 recombinase system. Glibenclamide can be taken in OATP1B3 (wild-type), OATP1B3 (344T > G) and OATP1B3 (699G > A)-HEK293T cells with the V_max_ values of 44.91 ± 7.97, 46.08 ± 8.69, and 37.31 ± 5.04 pmol/min/mg, while glipizide was taken in with V_max_ of 16.50 ± 3.64, 16.87 ± 4.23, and 13.42 ± 2.79 pmol/min/mg, respectively. The internal clearance of glibenclamide and glipizide in OATP1B3 (699G > A) was less than that in wild-type. Glibenclamide can be metabolized in CYP2C9*1, *2 and *3 recombinase system with the V_max_ values of 1.58 ± 0.71, 0.69 ± 0.25, and 0.41 ± 0.13 nmol/min/mg protein, while glipizide was metabolized with V_max_ of 8.82 ± 2.78, 5.99 ± 1.95, and 2.87 ± 1.03 nmol/min/mg protein, respectively. The internal clearance of glibenclamide and glipizide in CYP2C9*2 and *3 was markedly reduced compared to that in CYP2C9*1. These results collectively demonstrate that OATP1B3 (699G > A) and CYP2C9*2 and *3 have a significant influence on the transport and metabolism of glibenclamide and glipizide.

## Introduction

Glibenclamide, glipizide, gliclazide and glimepiride are commonly used hypoglycemic agents for the treatment of non-insulin-dependent type 2 diabetes mellitus. The pharmacokinetics and pharmacodynamics of these drugs in human often show inter-individual variability, which may be resulted from the genetic differences of metabolic enzymes and transporters in individuals^1,2^. Our previous studies have revealed that glibenclamide and glipizide are the substrates of organic anion-transporting polypeptide 1B3 (OATP1B3), whereas gliclazide and glimepiride are the substrates of OATP1B1^3^. It has also been proved that these hypoglycemic agents including glibenclamide, glipizide, gliclazide and glimepiride are mainly metabolized by cytochrome P450 2C9 (CYP2C9). OATP and CYP2C9 play a vital role in drug disposition and often exhibit gene polymorphisms that significantly affect the transport and metabolism of many clinical drugs^4–6^. We have demonstrated that OATP1B1*5 and *15 and CYP2C9*2 and *3 have a significant effect on the transport and metabolism of glimepiride and gliclazide^7^.

The human OATP1B3 (gene name *SLCO1B3*) belongs to a liver-enriched OATP subfamily that is predominantly expressed on the basolateral membrane domain of hepatocytes^8^. There are obvious genetic polymorphisms in the *SLCO* gene family that may affect the *in vivo* drug disposal process. The polymorphism genes of *SLCO1B3* related to OATP1B3 transport activity included 334T > G(S112A), 699G > A(M233I), 1564G > T (G552C) and 1748G > A (G583E)^9,10^. Sequence variation in genes encoding OATP1B3 has often been found to contribute significantly to inter-individual differences in the disposition of many clinically relevant drugs^11–13^. SLCO1B3 334T > G (Ser112Ala) and 699G > A (Met233Ile) were commonly reported to be associated with the altered transport function *in vitro* compared to the expressed wild-type protein. The recent studies have shown SLCO1B3 334T > G and 699G > A influence the pharmacokinetic profile of a newly identified substrate, the glucuronide metabolite of the immunosuppressant mycophenolic acid^14,15^.

CYP2C9 accounts for about 20% of the total cytochrome P450 in liver microsomes and takes part in the metabolism of approximately 10–20% of commonly used drugs^16,17^. There are marked polymorphisms in the human *CYP2C9* gene that significantly affect drug metabolism^18,19^. In Caucasians, the frequencies of the CYP2C9*2 (Arg 144Cys) and CYP2C9*3 (Ile359Leu) alleles of the CYP2C9 variant are 0.08–0.125, 0.03–0.085, respectively. But in the Asian population, CYP2C9*2 is rare and the allele frequencies of *3 and *13 (Leu90Pro) were 0.043–0.077 and 0.00–0.012, respectively^20,21^. Lots of *in vitro* and *in vivo* studies have shown that CYP2C9 *2 and CYP2C9 *3 point mutation have crucial impacts on CYP enzyme activity and significant influences on drug metabolism^22–25^.

It is unclear to date how OATP1B3 and CYP2C9 polymorphisms are associated with the substantial inter-individual variation of glibenclamide and glipizide in human. In the present study, therefore, we aimed to explore the effects of OATP1B3 and CYP2C9 polymorphisms on the transport and metabolism of glibenclamide and glipizide by using HEK293T cells expressing OATP1B3 and human recombinant enzymes expressing CYP2C9 with different genotypes.

## Materials and Methods

### Chemicals and reagents

Glibenclamide (purity 99.9%), glipizide (purity 99.8%), and gliquidone (purity 99.3%) were supplied by the National Institute for the Control of Pharmaceutical and Biological Products (Beijing, China). Dulbecco’s modified Eagle’s medium (DMEM), fetal bovine serum (FBS) and Hank’s balanced salt solution (HBSS) were provided by Solarbio Co., Ltd (Beijing, China). NADPH, 6-phosphate-glucose, and 6-p-glucose dehydrogenase were purchased from Solarbio Co., Ltd. Human CYP2C9*1, *2 and *3 recombinases (1nmol/ml P450, 10 mg/ml protein) were provided by the Research Institute for Liver Diseases (Shanghai Corporation Limited, Shanghai, China). Methanol and ethyl acetate were from Merck Co. Ltd (Darmstadt, Germany). All other chemicals and solvents were of the highest grade or analytical grade commercially available.

### Cell line

**Figure 1:**
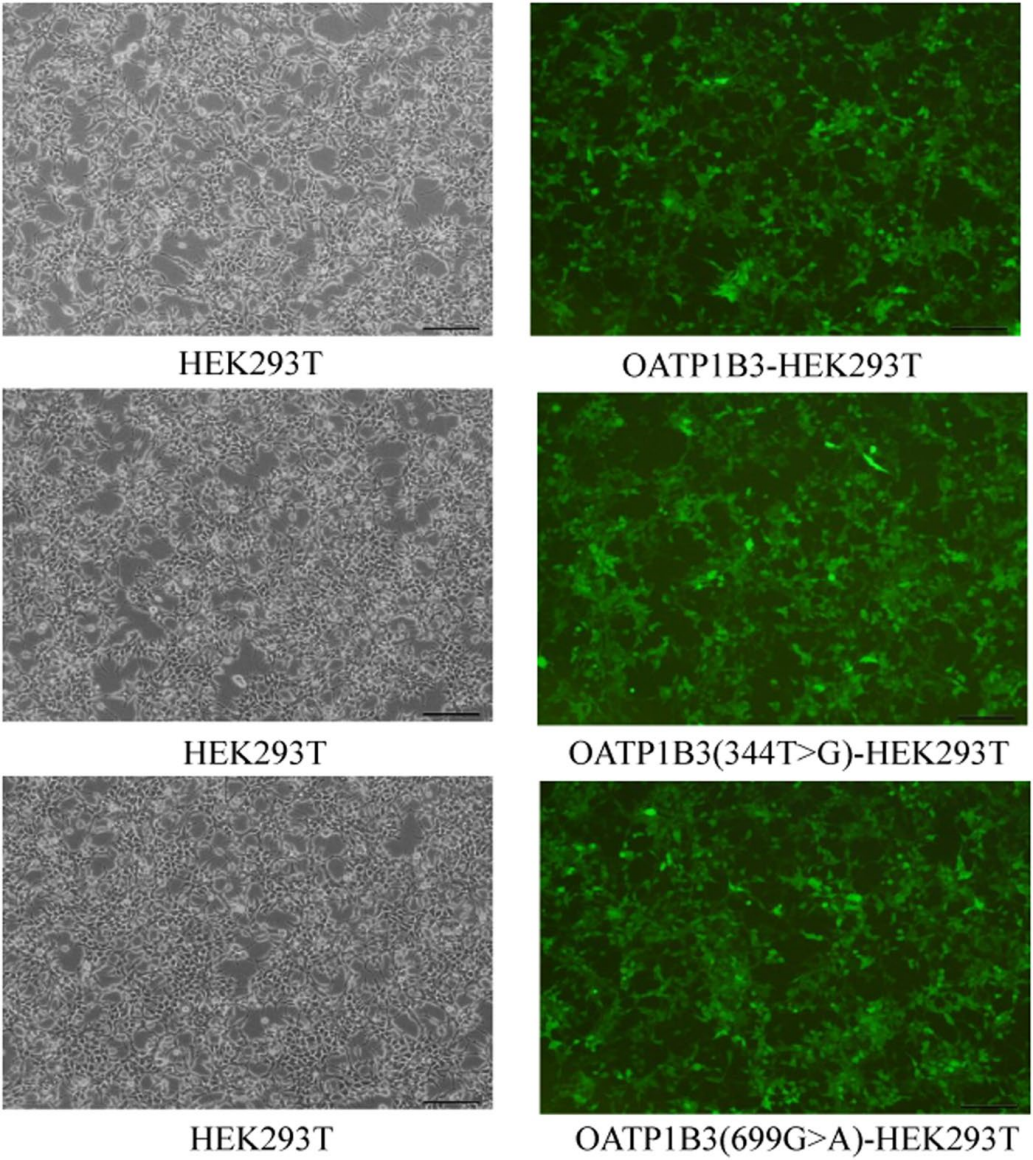
The fluorescent photos of HEK293T cells after being transfected with lentivirus including pGC-FU-OATP1B3, pGC-FU-OATP1B3 (344T > G) and pGC-FU-OATP1B3 (699G > A).

**Figure 2:**
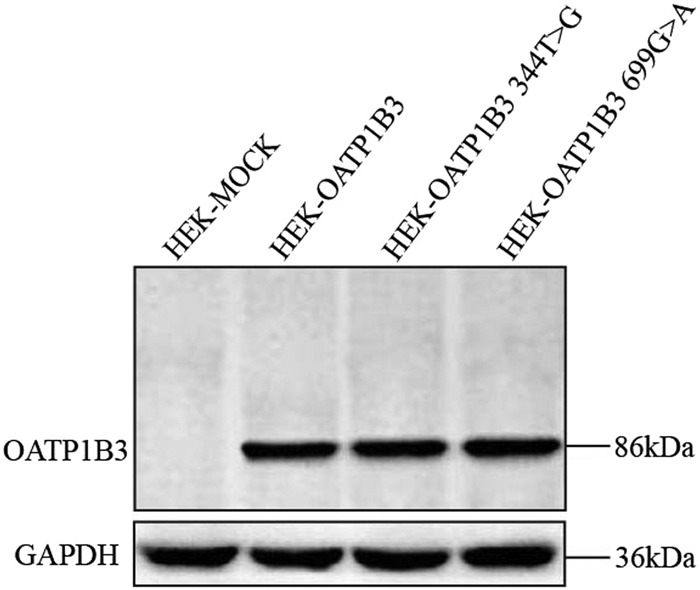
Western blot analysis of OATP1B3 in HEK-MOCK, HEK-OATP1B3, HEK-OATP1B3 (344T > G) and HEK-OATP1B3 (699G > A).

The HEK293T cells stably expressing OATP1B3, OATP1B3(344T > G) and OATP1B3(699G > A), respectively, were provided by Shanghai NOBOVIO Co., Ltd. (Shanghai, China). The green fluorescent protein (GFP) in HEK293T was observed using fluorescence microscope (Fig. 1). The expression of OATP1B3, OATP1B3 (344T > G) and OATP1B3 (699G > A) was detected by western blot (Fig. 2) and the relative quantity of OATP1B3 protein was calculated for the standardization. The HEK293T cells were cultured in high-glucose (4.5 g/L) DMEM with 10% FBS, 100 U/ml penicillin, and 100 μg/ml streptomycin at 37 °C under 5% CO_2_ humidified air.

### Western blot analysis

The OATP1B3 expression in OATP1B3, (344T > G), (699G > A)-HEK293T cells was detected by western blot. Cell extracts were prepared in lysis buffer. The cell debris was removed by centrifugation at 12,000 × g at 4 °C for 15 min and the total protein concentration was measured using a BCA Protein Assay Kit (Vazyme Biotech Co., Ltd, Nanjing, China). Protein samples (50 μg) were subjected to SDS-PAGE and electrophoretically transferred to PVDF membranes (EMD Millipore, Bedford, MA, USA). Immunoblots were probed with mouse polyclonal OATP1B3 antibody (diluted 1:5000) (Proteintech, Chicago, IL, USA) and with mouse polyclonal anti-GAPDH (diluted 1:4000) (Sigma Co., Ltd, USA) antibody as the loading control. After incubation with HRP-conjugated secondary antibody (Santa Cruz Biotechnology, Inc., Dallas, TX, USA), signals were detected by Super Signal West Dura (Pierce, Rockford, IL, USA) using a Bio-Rad ChemiDoc XRS imaging system (Bio-Rad Laboratories), and densitometry analysis was performed using Image Lab Sofware (Bio-Rad Laboratories).

### Quantification of glibenclamide and glipizide in HEK293T cells

Firstly, the cells were adjusted to an appropriate density (7.0 × 10^5^/ml) and then were added to 12-well culture plates at 1.0 ml per well. For time-dependent studies, the uptake of glibenclamide and glipizide in HEK293T cells were measured at 2, 5, 10, 15, 20, and 30 min. For concentration-dependent studies, the uptake of glibenclamide and glipizide in HEK293T cells were measured in a series of concentrations of 2.5, 5, 10, 20, 40, and 80 μM. Experiments contained MOCK-HEK293T cells group, OATP1B3-HEK293T cells group, OATP1B3 (344T > G)-HEK293T cells group and OATP1B3 (699G > A)-HEK293T cells group. Cells were incubated at 37 °C for 20 min with HBSS containing 10 mM HEPES (HBSS-HEPES, 99:1, v/v). At the end of the incubation period, indicated concentrations of glimepiride or gliclazide were added, cells were then placed in the incubator at 37 °C for 15 min or for specified times. The experiment was terminated by removing the medium and immediately washing cells with ice-cold HBSS four times and then adding 1 ml sterile water. Cells were disrupted by 4 cycles of freeze–thawing at −80 °C, and 200 μl of cell lysate was placed in a 1.5 ml centrifuge tube with 20 μl of 5 μM gliquidone internal standard, 30 μl of glacial acetic acid, and 800 μl ethyl acetate. After vortexing for 5 min, the tube was centrifuged at 15,000 × *g* for 10 min, and 700 μl of the supernatant was concentrated by vacuum drying at 55 °C for 30 min. The resulting concentrate was dissolved in 200 μl of acetonitrile, vortexed for 5 min, and centrifuged at 15,000 × *g* for 10 min, 100 μl of supernatant was finally used for LC/MS analysis^3^. The protein concentration of each sample was determined using a BCA Protein Assay Kit (Vazyme Biotech Co., Ltd, Nanjing, China). For the standardization of the OATP1B3 protein content in HEK293T cells, the total protein content (determined by BCA Protein Assay Kit) was divided by OATP1B3, OATP1B3 (344T > G), OATP1B3 (699G > A) relative protein content (determined by western blot).

The concentrations of glibenazide and glipizide in cell samples were determined using an LC-MS system equipped with Shimadzu LC-20AB pumps and a 2010EV mass spectrometer (Shimadzu Corporation, Kyoto, Japan). The separation was performed on an C_18_ column (150 mm × 2.0 mm, 5 μm) with the mobile phase consisting 0.1% formic acid (A) and acetonitrile (B) at a flow rate of 0.2 ml/min (A:B = 30:70). The flow rates of the drying gas and nebulizing gas were set to 2.0 L/min and 1.5 L/min, respectively. The heating block temperature was 200 °C, and the temperature of the solvent removal device was 250 °C. The CDL voltage was 250 V, and the detection voltage was 1.85 kV. The analysis was performed in selective ion monitoring mode at [M-H]^−^ *m/z* = 528.25 for gliquidone (internal standard), [M-H]ˉ *m/z* = 494.25 for glibenclamide, and [M-H]^−^ *m/z* = 446.30 for glipizide. The retention time of glibenclamide, glipizide and gliquidone was 3.5 min, 2.5 min and 5.0 min, respectively^3^.

### Quantification of glibenclamide and glipizide in CYP2C9 recombinant enzyme incubation system

The final volume of incubation system was 200 μl, containing 50 mM sodium phosphate buffer (pH = 7.4) and 0.4 mg/ml human recombinant CYP2C9 (CYP2C9*1, CYP2C9*2, and CYP2C9*3). A series of concentrations of glibenclamide and glipizide (1–80 μM) were prepared in methanol, and the final concentration of methanol was no more than 0.5% (v/v). The reactions took place at 37 °C in a shaker. After pre-warming for 5 min, the reaction was initiated by adding a NADPH regenerating system containing 1.3 mM NADP^+^, 3.3 mM glucose-6-phosphate, 3.3 mM MgCl_2_, and 0.4 U/ml glucose-6-phosphate dehydrogenase. In the negative controls, the NADPH regenerating system was not added. The reactions were stopped at 30 min by adding 100 μl ice-cold acetonitrile. Samples were vortexed and diluted to the proper concentrations, and 20 μl of 5 μM gliquidone (IS) was added to each sample. The samples were then vortexed for 5 min at room temperature and centrifuged at 15,000 × *g* for 10 min. Finally, 100 μl of each reconstituted sample was used for LC/MS analysis^7^.

### Statistical analysis

All data are presented as means with their standard deviations (mean ± SD). Kinetic parameters were obtained using GraphPad Prism (GraphPad Software 5.01, San Diego, CA, US) followed by non-linear, least-squares regression analysis via the Michaelis–Menten equation. Statistical analysis was conducted using SPSS 12.0. A one-way analysis of variance (ANOVA) was performed to determine whether the differences among all the groups were statistically significant, and then Dunnett’s post hoc test was carried out to statistically. *P* < 0.05 was considered to be statistically significant.

## Results

### Uptake of glibenclamide and glipizide in OATP1B3-HEK293T cells

**Figure 3:**
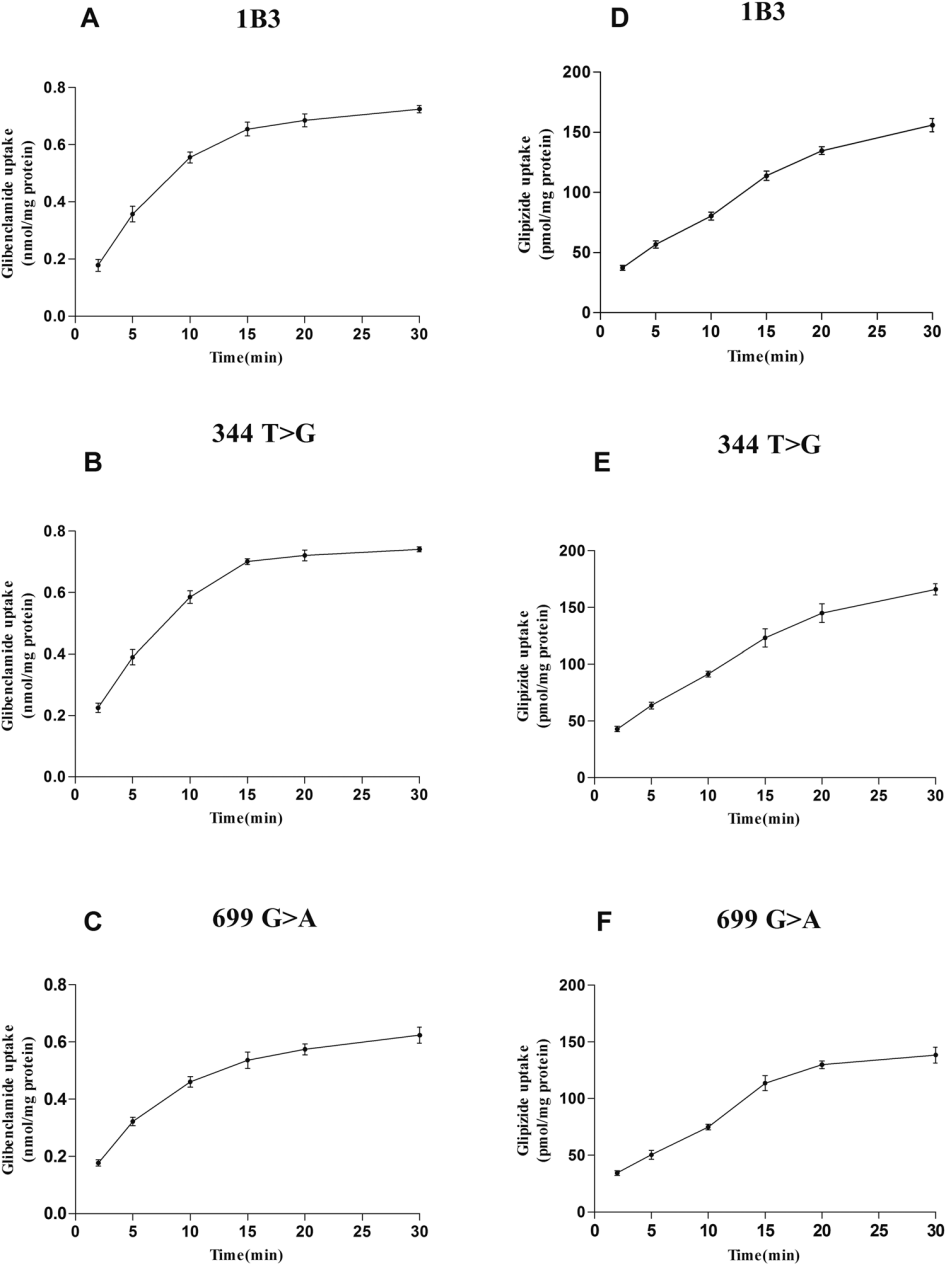
Time-dependent uptake of glibenclamide (**A**–**C**) and glipizide (**D**–**F**) in OATP1B3, (344T > G), (699G > A)-HEK293T cells. Forty micromolar glibenclamide and glipizide were incubated with OATP1B3, (344T > G), and (699G > A)-HEK293T cells for 2, 5, 10, 15, 20, or 30 min. Data points represents the mean ± SD of three separate experiments.

**Figure 4:**
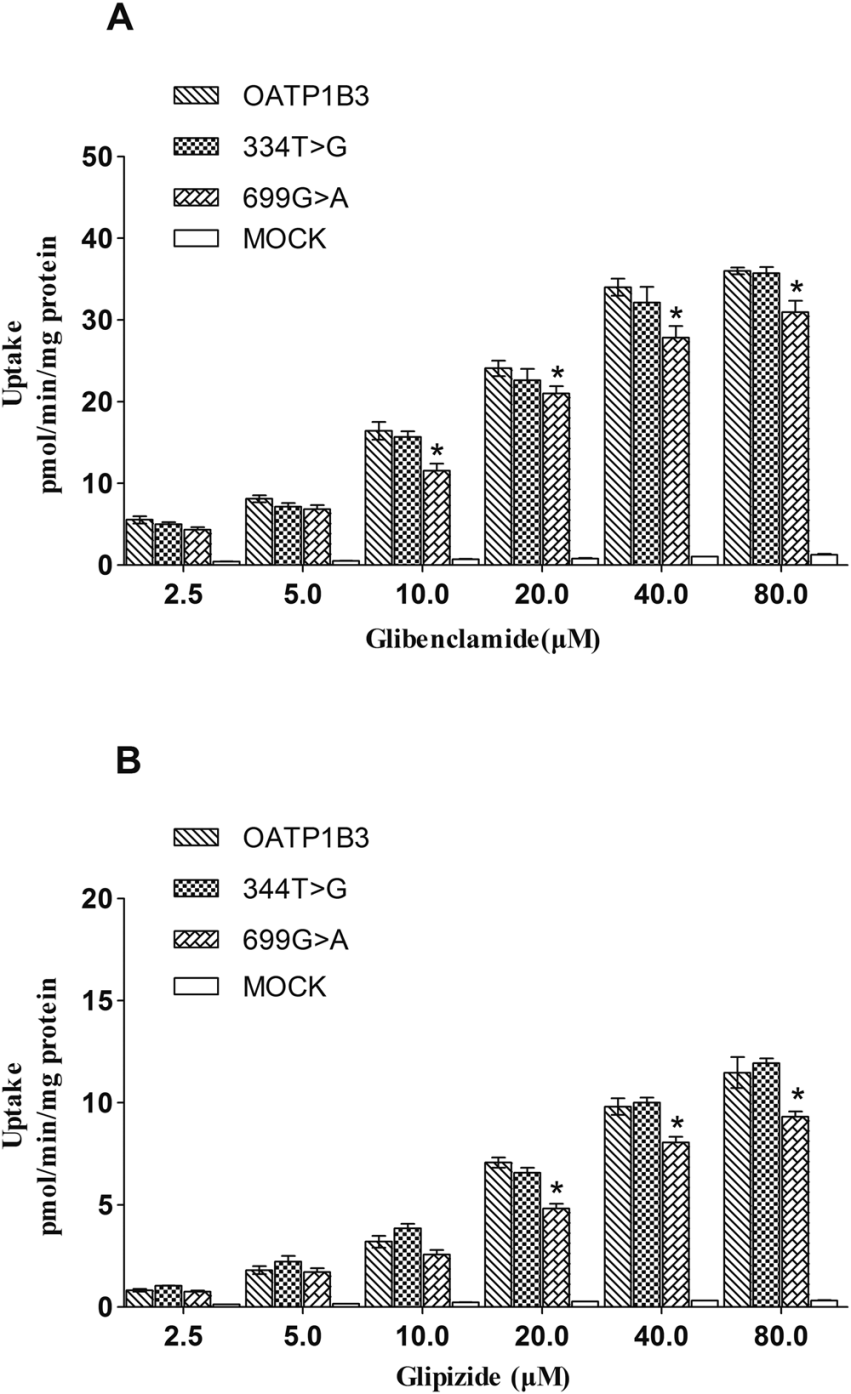
Concentration-dependent uptake of glibenclamide (**A**) and glipizide (**B**) in OATP1B3, (344T > G), (699G > A)-HEK293T cells. A series of concentrations of glibenclamide and glipizide (2.5, 5, 10, 20, 40, 80 μM) were incubated with OATP1B3, (344T > G), (699G > A) and MOCK-HEK293T cells for 15 min. Data points represents the mean ± SD of four separate experiments.

**Figure 5:**
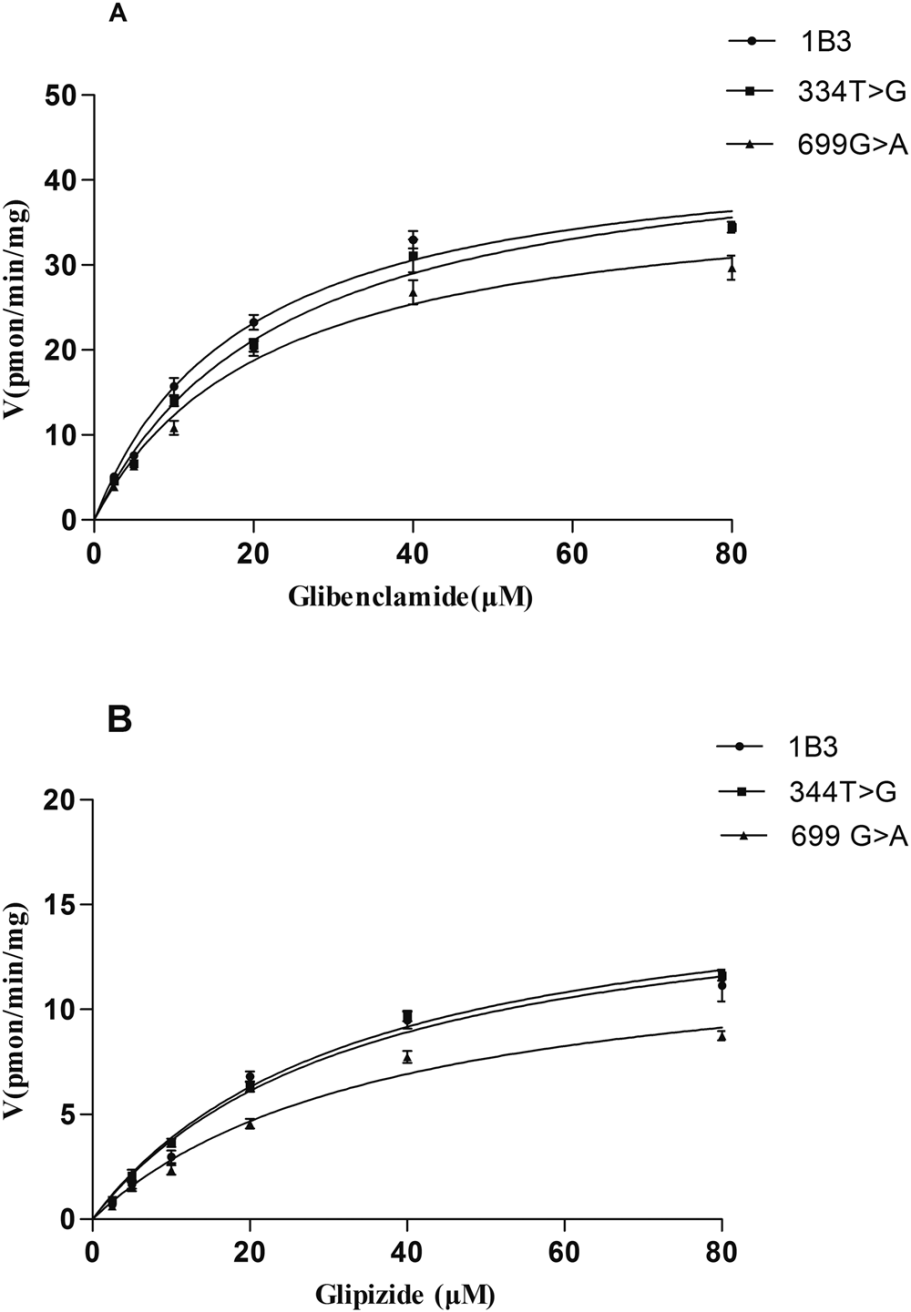
Kinetics of glibenclamide (**A**) and glipizide (**B**) in OATP1B3, (344T > G), and (699G > A)-HEK293T cells. Transport kinetics of glibenclamide (**A**) and glipizide in OATP1B3, (344T > G), and (699G > A)-HEK293T cells was conducted for 15 min at varying concentrations of glibenclamide and glipizide (2.5–80 μM). The data was obtained after the subtraction of MOCK-HEK293T value. Parameters for saturation kinetics (V_max_ and K_m_) were estimated by nonlinear curve fitting using Prism (GraphPad Software). Data are expressed as mean ± SD of four separate experiments.

Uptake experiments of glibenclamide and glipizide were carried out as described with the addition of different concentrations of glibenclamide and glipizide (2.5–80 μM). The time-dependent experiments showed that the uptake of glibenclamide and glipizide nearly attained a steady-state at 15 min (Fig. 3). Concentration-dependent experiments showed that the uptake of glibenclamide and glipizide in OATP1B3-HEK293T cells was nearly saturated at 40 μM (Figs 4 and 5). The transport of glibenclamide and glipizide by OATP1B3 was related to the genotype. Compared with the wild-type OATP1B3, mutants OATP1B3 (699G > A) showed a decreased transport capacity, while mutants OATP1B3 (344T > G) had no influence on the transport capacity. The uptake of glibenclamide and glipizide in OATP1B3 (699G > A)-HEK293T cells was less than that in OATP1B3-HEK293T cells (Fig. 4). For glibenclamide, the V_max_ and intrinsic clearance (CL_int_, defined as V_max_/K_m_) in OATP1B3 (699G > A)-HEK293T cells was decreased to 85% and 65% of that in OATP1B3-HEK293T cells, respectively (Fig. 5, Table 1). For glipizide, the V_max_ and CL_int_ in OATP1B3 (699G > A)-HEK293T cells was decreased to 80% and 64% of that in OATP1B3-HEK293T cells, respectively (Fig. 5, Table 2).

**Table 1:** Uptake kinetic parameters of glibenclamide in different genotypes OATP1B3 expression of HEK293T cells (n = 4, Mean ± SD).

Genotype	K_m_ (μM)	V_max_ (pmol/min /mg)	CL_int_
OATP1B3	18.91 ± 5.31	44.91 ± 7.97	2.39 ± 1.06
344T > G	21.55 ± 3.06	46.08 ± 8.69	2.13 ± 0.84
699G > A	25.91 ± 6.79*	37.31 ± 5.04*	1.51 ± 0.70*

**p < 0.01, *p < 0.05 (*vs* OATP1B3 group), CL_int_ (intrinsic clearance) = V_max_/K_m_.

**Table 2:** Uptake kinetic parameters of glipizide in different genotypes OATP1B3 expression of HEK293T cells (n = 4, Mean ± SD).

Genotype	K_m_ (μM)	V_max_ (pmol/min/mg)	CL_int_
OATP1B3	28.02 ± 6.91	16.50 ± 3.64	0.59 ± 0.16
344T > G	30.52 ± 5.36	16.87 ± 4.23	0.55 ± 0.24
699G > A	33.55 ± 6.63	13.42 ± 2.79*	0.38 ± 0.17*

**p < 0.01, *p < 0.05 (*vs* OATP1B3 group), CL_int_ (intrinsic clearance) = V_max_/K_m_.

### Metabolism of glibenclamide and glipizide by recombinant CYP2C9

**Figure 6:**
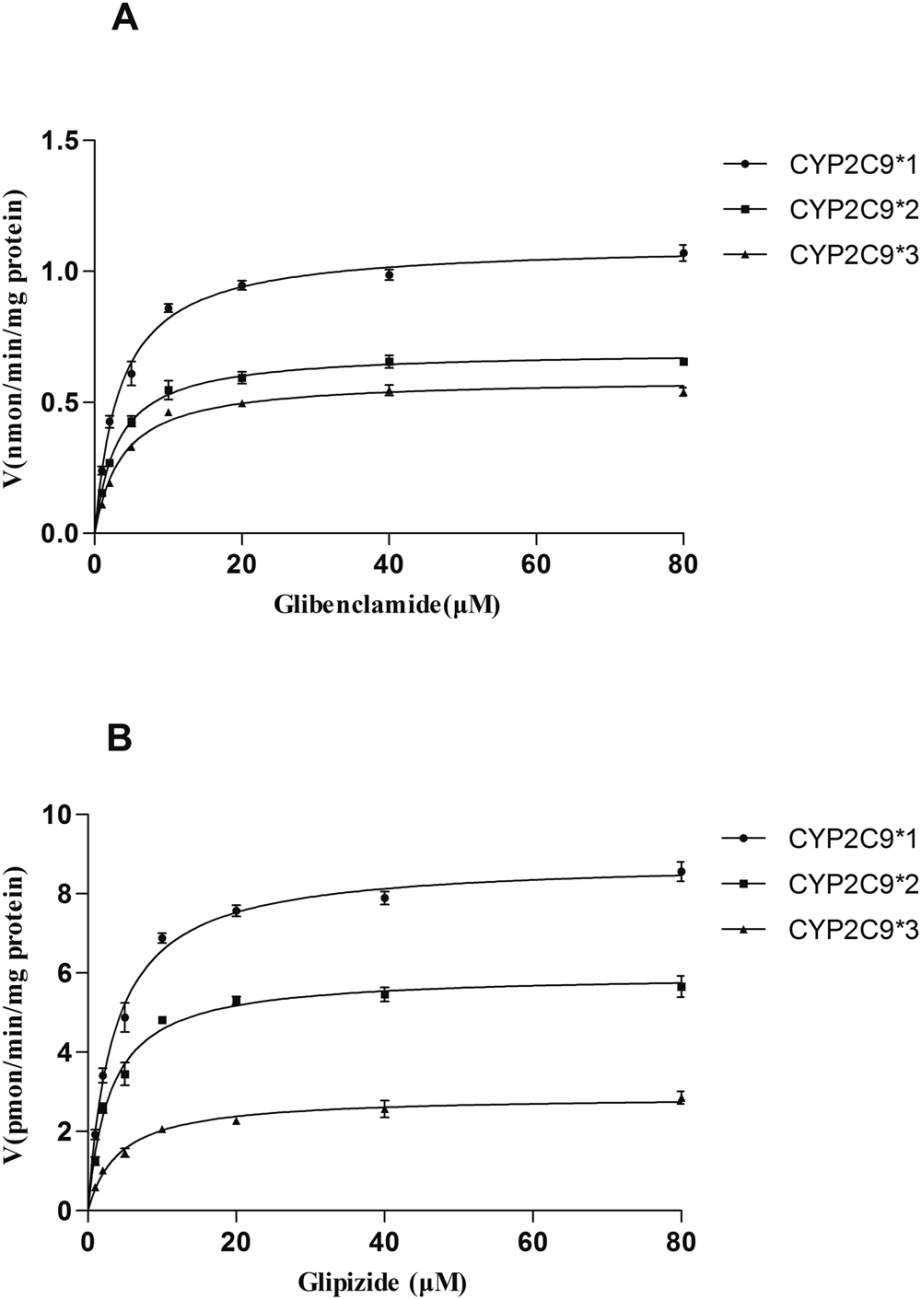
Michaelis–Menten curves of enzymatic activity of recombinant CYP2C9*1,*2, and *3 towards glibenclamide (**A**) and glipizide (**B**). Michaelis–Menten curves of the enzymatic activity in CYP2C9*1,*2, and *3 recombinases were studied at varying concentrations of glibenclamide and glipizide (1, 2, 5, 10, 20, 40, 80 μM). Data points represent the mean ± SD of three separate experiments.

The catalytic activities of wild-type CYP2C9*1 and two allelic variants CYP2C9*2 and *3 were assessed by using glibenclamide and glipizide as substrates. Michaelis–Menten plots for each of the CYP2C9 variants are shown in (Fig. 6), and the corresponding kinetic parameters are summarized in (Tables 3 and 4). As shown in (Tables 3 and 4), the two variants exhibited changed V_max_ and CL_int_ values of glibenclamide and glipizide as compared to the wild-type protein. The two variants, CYP2C9*2 and CYP2C9*3, caused the V_max_ of glibenclamide decreased to 43% and 25%, respectively and the CL_int_ of glibenclamide decreased to 44% and 26%, respectively, compared with the wild-type (Table 3). For glipizide, the V_max_ was decreased to 68% and 32%, respectively, and the CL_int_ was decreased to 55% and 23% respectively, in CYP2C9*2 and *3 recombinant compared to CYP2C9*1 recombinant as shown in (Table 4). These results revealed that point mutations in CYP2C9*2 and CYP2C9*3 had vital impacts on CYP enzyme activity and significantly affected the metabolism of glibenclamide and glipizide.

**Table 3:** Glibenclamide metabolic kinetic parameters in human recombinase CYP2C9*1/*2/*3 (n = 3, mean ± SD).

Protein	K_m_ (μmol/L)	V_max_ (nmol/min/mg)	CL_int_
CYP2C9*1	3.17 ± 1.02	1.58 ± 0.71	0.45 ± 0.12
CYP2C9*2	3.46 ± 1.56	0.69 ± 0.25**	0.20 ± 0.17**
CYP2C9*3	3.90 ± 2.34*	0.41 ± 0.13**	0.12 ± 0.08**

**p < 0.01, *p < 0.05 (*vs* CYP2C9*1 group), CL_int_ (intrinsic clearance) = V_max_/K_m_.

**Table 4:** Glipizide metabolic kinetic parameters in human recombinase CYP2C9*1/*2/*3 (n = 3, mean ± SD).

Protein	K_m_ (μmol/L)	V_max_ (nmol/min/mg)	CL_int_
CYP2C9*1	3.07 ± 1.31	8.82 ± 2.78	2.95 ± 1.61
CYP2C9*2	3.51 ± 1.76	5.99 ± 1.95*	1.69 ± 0.47*
CYP2C9*3	4.10 ± 2.66*	2.87 ± 1.03**	0.72 ± 0.48**

**p < 0.01, *p < 0.05 (*vs* CYP2C9*1 group), CL_int_ (intrinsic clearance) = V_max_/K_m_.

## Discussion

OATP1B3 is a liver-specific transporter that has an important role in transporting certain endogenous and exogenous substrates into the liver. Consequently, this protein is suspected to play a central function in the disposition of drugs for both metabolism and hepatobiliary elimination^26,27^.

OATP1B3 is encoded by the (SLCO) 1B3 gene and several SLCO1B3 nucleotide sequence variations have been identified. The OATP1B3 gene polymorphisms T334G (Ser112Ala) and G699A (Met233Ile) show a complete linkage disequilibrium with an allele frequency of 0.728. (112Ala/233Ile), the linkage disequilibrium of these mutants will lead to a decrease in OATP1B3 transport activity^9^. Changes in the transport function of OATP1B3 caused by mutation affect the transfer of substrate drugs, which eventually changes the blood drug concentration of a drug and affects drug treatment^28,29^. Two common variants, SLCO1B3 334T > G (Ser112Ala) and 699G > A (Met233Ile), were found to be associated with altered transport function *in vitro* compared to the expressed wild-type protein. Schwarz *et al*.^9^. found that the translocation of cholecystokinin-8 (CCK8) and rosuvastatin in the highly expressed OATP1B3-Met233Ile (699G > A) HeLa cells was significantly lower than that in wild-type OATP1B3 HeLa cells.

As the substrate for the uptake of OATP1B3, glibenclamide and glipizide, the blood plasma concentration may be influenced by OATP1B3 genetic polymorphism. In the present study, we found that the uptake of glibenclamide and glipizide in OATP1B3 (699G > A)-HEK293T cells was significantly reduced compared with OATP1B3-HEK293T cells (Fig. 5). For glibenclamide in OATP1B3 (699G > A) -HEK293T cells, the V_max_ was decreased to 85% and CL_int_ was decreased to 65% of that in OATP1B3-HEK293T cells. For glipizide, the V_max_ was decreased to 80% and CL_int_ was decreased to 64%. Thus, it can be deduced that the uptake of glibenclamide and glipizide are affected by mutation 699G > A. However, the mutants OATP1B3 (344T > G) had no influence on the transport capacity. Further *in vivo* studies are needed to perform in the future for confirming the *vitro* results.

It is well-known that metabolic activity changes due to CYP2C9 polymorphisms play an important role in adverse drug reactions. The changes of CYP2C9 activity may increase the risk of adverse drug reactions for patients when they are administered with CYP2C9-specific substrates such as S-warfarin, phenytoin, glipizide, and tolbutamide which have narrow therapeutic windows^30^. Niemi *et al*.^25^. reported that the AUC of glibenclamide in individuals heterozygous for the CYP2C9*3 allele (n = 52) was 280% of the respective value in the CYP2C9*1/*1 subjects (n = 55). Yin *et al*.^22^. also demonstrated that the oral plasma AUC of glibenclamide in the CYP2C9*1/*3 subjects (n = 56) of the Chinese population was increased by approximately 100% compared with that in the CYP2C9*1/*1 subjects. These clinical studies appear to suggest that CYP2C9 contributes significantly to glibenclamide metabolism *in vivo*. At the some time, glipizide oral clearance in one homozygous carrier of the CYP2C9*3 allele was only 20% of that in carriers of the CYP2C9 wild-type^31^. *In vivo* studies have shown that CYP2C9 gene polymorphism has a significant effect on the sulfonylurea metabolism. However, *in vivo* studies are often affected by many other factors, such as physiology, age, and food. Of note, the significant changes in metabolism associated with the CYP2C9*2 and *3 alleles demonstrated *in vitro* and *in vivo* for a variety of substrates do not appear to be consistent for all substrates evaluated^19,32^. Therefore, we study the impact of CYP2C9 gene polymorphisms of drug metabolism by recombinant enzyme *in vitro* model. In the present study, we found that CYP2C9*2 was less efficient than CYP2C9*1 in metabolizing glibenclamide and glipizide. However, this was not due to differences in the apparent K_m_, but was the result of a decreased V_max_ and CL_int_ in the metabolism of glibenclamide and glipizide by CYP2C9*2 when compared with CYP2C9*1. Similarly, the allelic variant CYP2C9*3 was also less efficient than CYP2C9*1 in metabolizing glibenclamide and glipizide. This may be explained by the increased K_m_ and decreased V_max_ in the metabolism of glibenclamide and glipizide by CYP2C9*2 (Table 4). It is possible that CYP2C9*2 and CYP2C9*3 are risk factors for adverse drug reactions such as hypoglycemia or allergic reactions due to sulphonylurea.

In summary, glibenclamide and glipizide can be transported by OATP1B3 and metabolized by CYP2C9. The mutants OATP1B3 (699G > A) can significantly decrease their transport capacity, while CYP2C9*2 and *3 mutants show significantly reduced metabolism toward glibenclamide and glipizide. Taken together with our previous results^7^ that the mutations of OATP1B1*5, *15 and CYP2C9*2, *3 significantly affect the transport and metabolism of glimepiride and gliclazide, it can be deduced that OATP1B1/1B3 and CYP2C9 play a vital role in the disposition of these oral hypoglycemic agents in humans and their pharmacokinetic behaviors can be markedly influenced by OATP1B1/1B3 and CYP2C9 gene polymorphisms. We should pay much attention to the potential impacts of OATP1B1/1B3 and CYP2C9 gene polymorphisms on the pharmacokinetics and pharmacodynamics when using these oral hypoglycemic drugs in clinical settings.
